# Developing Pulmonary Rehabilitation for COVID-19: Are We Linked with the Present Literature? A Lexical and Geographical Evaluation Study Based on the Graph Theory

**DOI:** 10.3390/jcm10245763

**Published:** 2021-12-09

**Authors:** Augusto Fusco, Luca Padua, Daniele Coraci, Claudia Loreti, Letizia Castelli, Cosimo Costantino, Antonio Frizziero, Elisabetta Serafini, Lorenzo Biscotti, Roberto Bernabei, Silvia Giovannini

**Affiliations:** 1 UOC Neuroriabilitazione ad Alta Intensità, Fondazione Policlinico Universitario A. Gemelli IRCCS, 00168 Rome, Italy; augusto.fusco@policlinicogemelli.it (A.F.); luca.padua@unicatt.it (L.P.); letizia.castelli@gmail.com (L.C.); 2 Department of Geriatrics and Orthopaedics, Università Cattolica del Sacro Cuore, 00168 Rome, Italy; roberto.bernabei@unicatt.it (R.B.); silvia_giovannini@yahoo.it (S.G.); 3 Physical Medicine and Rehabilitation Unit, Department of Neurosciences, University of Padova, 35122 Padua, Italy; 4 Department of Aging, Neurological, Orthopaedic and Head-Neck Sciences, Fondazione Policlinico Universitario A. Gemelli IRCCS, 00168 Rome, Italy; claudia.loreti@policlinicogemelli.it (C.L.); elisabetta.serafini@policlinicogemelli.it (E.S.); lorenzo.biscotti@unicatt.it (L.B.); 5 Department of Medicine and Surgery, University of Parma, 43121 Parma, Italy; cosimo.costantino@unipr.it (C.C.); antonio.frizziero@unipr.it (A.F.); 6 Presiding Officer of Geriatric Care Promotion and Development Centre (C.E.P.S.A.G), Università Cattolica del Sacro Cuore, 00168 Rome, Italy; 7 UOS Riabilitazione Post-Acuzie, Fondazione Policlinico Universitario A. Gemelli IRCCS, 00168 Rome, Italy

**Keywords:** COVID-19, SARS-CoV-2, rehabilitation, graph theory, personalized medicine, interstitial lung disease

## Abstract

The Coronavirus Disease 2019 (COVID-19) pandemic is a severe ongoing global emergency. Despite high rates of asymptomatic patients, in many cases, the infection causes a rapid decline in pulmonary function due to an acute respiratory distress-like syndrome, leading to multi-organ failure and death. To date, recommendations about rehabilitation on COVID-19 are based on clinical data derived from other similar lung diseases. Rehabilitation literature lacks a standard taxonomy, limiting a proper evaluation of the most effective treatments for patients after COVID-19 infection. In this study, we assessed the clinical and rehabilitative associations and the geographical area involved in interstitial lung diseases (ILD) and in COVID-19, by a mathematical analysis based on graph theory. We performed a quantitative analysis of the literature in terms of lexical analysis and on how words are connected to each other. Despite a large difference in timeframe (throughout the last 23 years for ILD and in the last 1.5 years for COVID-19), the numbers of papers included in this study were similar. Our results show a clear discrepancy between rehabilitation proposed for COVID-19 and ILD. In ILD, the term “rehabilitation” and other related words such as “exercise” and “program” resulted in lower values of centrality and higher values of eccentricity, meaning relatively less importance of the training during the process of care in rehabilitation of patients with ILD. Conversely, “rehabilitation” was one of the most cited terms in COVID-19 literature, strongly associated with terms such as “exercise”, “physical”, and “program”, entailing a multidimensional approach of the rehabilitation for these patients. This could also be due to the widespread studies conducted on rehabilitation on COVID-19, with Chinese and Italian researchers more involved. The assessment of the terms used for the description of the rehabilitation may help to program shared rehabilitation knowledge and avoid literature misunderstandings.

## 1. Introduction

The novel pandemic of coronavirus disease (COVID-19) caused by Severe Acute Respiratory Syndrome Coronavirus 2 (SARS-CoV-2), originated in 2019 in Wuhan and subsequently spread worldwide. Its impact has been devastating due to the high morbidity and mortality, resulting in a modification of people’s daily life due to the restrictions that governments around the world introduced to limit social interaction and diffusion of the virus [1,2].

Most studies conducted on COVID-19 have focused on acute care, epidemiology, viral structure, molecular mechanisms of infection, pathophysiology, diagnosis, clinical characteristics, and therapeutic options, including vaccination [3]. Later on, the emerging awareness of the consequences of COVID-19 disease has allowed rehabilitation to assume an increasingly important role. For this reason, research about COVID-19 rehabilitation has seen a rapid growth, however most of the studies were based on experiences in acute care settings or provided only general recommendations [4,5,6,7,8]. Nevertheless, these studies have been useful in planning the early rehabilitation treatments during the acute phase of the pandemic.

At present, respiratory rehabilitation is considered an essential strategy in the management of patients with COVID-19 [6,7,8,9,10,11,12], however studies are mostly based on other respiratory diseases’ guidelines (e.g., chronic obstructive pulmonary disease, interstitial lung disease, or asthma) rather than on specific protocols for COVID-19 [13,14,15]. Randomized and controlled trials on rehabilitation in patients with COVID-19 have been difficult to conduct due to the novelty of this disease and its acute impact on the health systems in the last two years throughout the world.

To our knowledge, only a small trial has been conducted in a group of elderly patients with COVID-19, showing an improvement in respiratory function, quality of life, and anxiety after 6 weeks of respiratory rehabilitation [16]. No data are yet available for the long-term management of these patients.

Rehabilitation literature lacks a standard taxonomy for the existing therapies, and this may limit a proper evaluation of the most effective treatments for patients after COVID-19 infection. This problem has been already documented in other studies in which authors analyzed the way researchers approach the bibliographic search [17,18,19]. Due to the vast number of sources and data related to research topics, researchers are asked to evaluate with particular attention the huge volume of studies, confirming the available information [20]. To overcome the possible lack of clarity, a quantitative analysis of the literature could be useful, especially in terms of lexical analysis and how words are connected to each other [21]. 

We performed a literature analysis on rehabilitation in patients with interstitial lung disease (ILD) and with COVID-19, using a method focused on the evaluation of the lexicon used in the scientific papers. In this way, we aimed to provide useful information to understand the evidence of literature and the connection between the main words used in the rehabilitation scientific literature for these diseases. We also assessed the geographical areas involved in the rehabilitation research.

## 2. Literature Evaluation 

To represent the literature evidence on the role of pulmonary rehabilitation in ILD and COVID-19, we have used a specific method based on the graph theory to evaluate the relationships between the scientific papers and specific words [20,21]. The graph theory is a mathematical analysis that visualizes the studied elements (defined as nodes: the number of papers and the words in our study) and their connections (defined as edges). The described method allows the production of a mathematical structure defined as the LExical Network analyzed by the Graph THeory (LENGTH) approach, assessing the impact of specific words in the literature. Additionally, this method can permit statistical analyses indicating the strength of the nodes into a specific topic. Our analysis considers the titles and the abstracts of papers found in PubMed and not the full text. We have already tested the same method in analogous studies for different diseases [17,18,19].

### 2.1. Lexicon Analysis

Our literature search was conducted, on 29 July 2021, on PubMed and PubMed Central, using the following string: “interstitial lung disease AND rehabilitation” and then “COVID-19 AND rehabilitation”. We focused our study on interstitial lung disease (ILD) due to its specific clinical features characterized by the injury to the alveolar epithelium and diminished pulmonary reserve and impaired gas exchange [22]. Even if different nosological entities, patients with ILD may present an increased rate of complications and death from COVID-19 [23].

No article-type filters were applied. The results (titles and abstracts of the papers) were exported, and the frequencies of the specific words used in these texts were calculated by the freeware software TXM 0.8.0 [24]. We selected the most frequent (present > 20 times) substantives and adjectives related to the topic and we calculated which papers contained these words in their title and abstract and how many times. A specific matrix was built and imported in the free software Gephi 0.9.2 to create a graph representing the connections between the papers and the keywords [25]. 

The analysis of the years of publication revealed that the 152 papers about ILD were published throughout a period of 23 years, with a gap in the first years of the third millennium. Conversely, the 134 papers about COVID-19 were published in the last two years. The highest dimensions of the nodes (corresponding to the degree) underlined the most frequent words used in the selected papers. The position in the center of the graph was related to the presence of many connections of the selected words for the number of involved papers.

**Figure 1:**
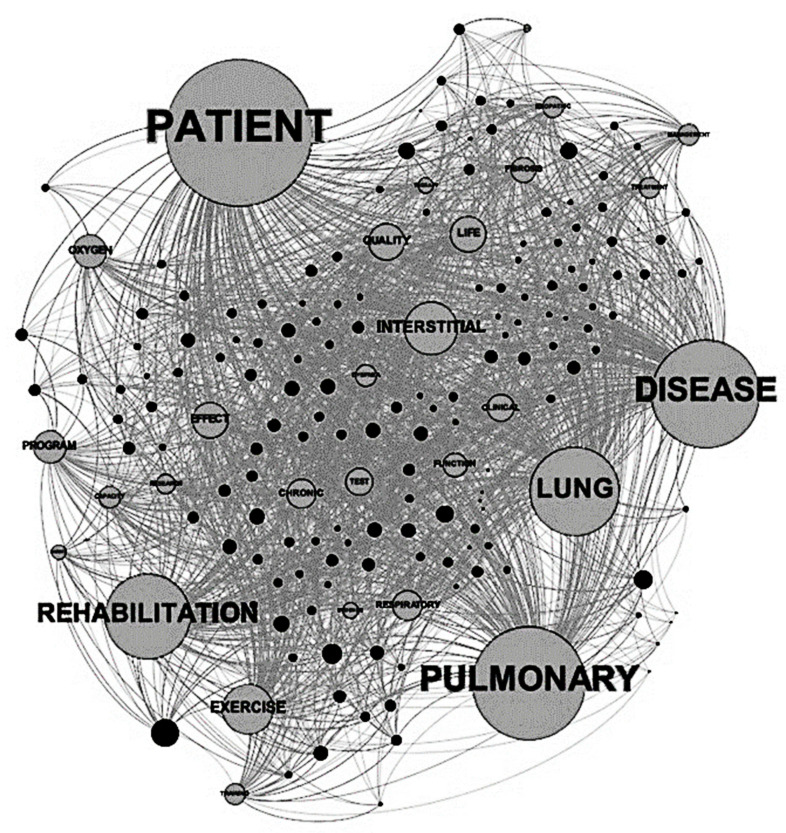
The graphical matrix of the connections between words and papers. The dimension of the nodes represents the number of citations (degree). The dimension of the edges is unique, even in cases of multiple connections between a word and the same paper. The layout used is ForceAtlas2. The nodes are the circles representing the searched words, and the papers are the black points. The edges represent the connections between a word and the papers.

The most frequent words in the lexicon analysis for ILD and pulmonary rehabilitation are shown in Figure 1. Recalculating the data by the real number of connections, the most cited words were “patient(s)” followed by “disease”, “pulmonary”, and “lung”, highlighting the clinical significance also during the rehabilitative training. The term “rehabilitation” was located in the third quartile. Despite the relatively small number of connections, “rehabilitation” resulted in low values of centrality (a measure linked to the importance of the element). From a graphical analysis of the figure, other words potentially related to rehabilitation were “exercise” and “program”, presenting an almost similar frequency (exercise more than program). In the graph, the term “exercise” was relatively close to the term “rehabilitation”, but its value of eccentricity was the highest, indicating a relatively high distance from the other nodes. Additionally, this word was connected with few papers, and in some of the studies, the term “rehabilitation” was not used. The term “function”, although with a similar degree to “exercise”, had a higher value of centrality and lower level of eccentricity, indicating a stronger connection with the other nodes. Other terms connected to rehabilitation were “effect”, “chronic”, “research”, and “capacity”. Finally, in the graph, little groups of nodes more related to the patient’s clinical features and their association with treatment included “quality” and “life” and partially “idiopathic, “treatment”, “management”, and “fibrosis”.

**Figure 2:**
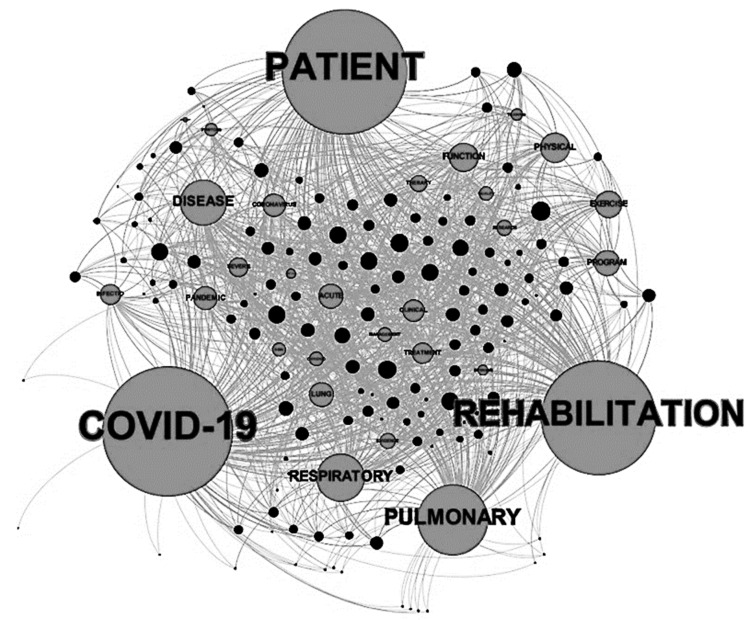
The graph of the connections between words and papers related to COVID-19 and pulmonary rehabilitation.

In the graph obtained from the analysis of COVID-19 and pulmonary rehabilitation literature, derived by a search limited to 1 January 2020 until 29 July 2021, we observed a different trend, as shown in Figure 2.

Recalculating by the real number of connections, the most cited words were always “patient(s)” followed in this case by “COVID-19” and “rehabilitation”. The term “pulmonary” was located in the third quartile as well as “disease”. The words more associated with the word rehabilitation (“exercise”, “physical”, and “program”) all presented a similar frequency. Then, in this case, the “function” was related more to the rehabilitation meaning of the word rather than to the pulmonary physiology. All these words were strongly connected, even if the number of papers was still low. The other connected terms were more associated with the nature of this new disease with a general meaning and a poor connection determined by a low level of centrality and power of nodes (e.g., “acute”, “pandemic”, “infectious“, “coronavirus”, and “clinical”).

### 2.2. Geographical Analysis

**Figure 3:**
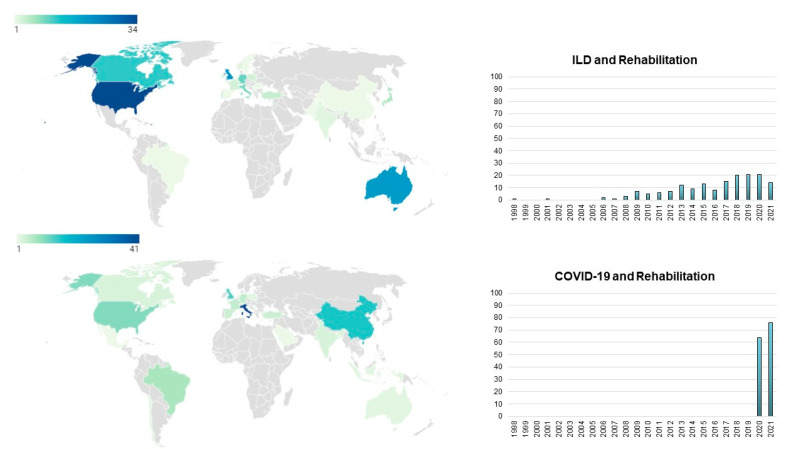
The histograms on the right indicate the number of papers published between 1998 and 2021 for interstitial lung disease and rehabilitation (above) and COVID-19 and rehabilitation (below). On the left, the geographical distribution of the papers. Colors indicate the number of papers per Country.

Data on the geographical origin of each paper were collected based on the country shown in the affiliation of the first author. We found the USA as the most productive country in terms of scientific literature about ILD, followed by the United Kingdom and Australia. With regard to COVID-19 papers, Italy was the country with the highest number of scientific publications, with China and the United Kingdom occupying the second and the third positions, respectively (Figure 3).

## 3. Discussion

Our study analyzed the available scientific papers on pulmonary rehabilitation for interstitial lung disease and COVID-19. We used the LENGTH method, which is a quantitative mathematical analysis based on graph theory, focused on the scientific literature lexicon. The LENGTH method is a new approach to develop a scientific network, recently proposed for the analysis of big data and disease [17,18,19,21,26]. Graph theory is a well-known method currently used for analyzing the data connection by neuroimaging or neurophysiology [26,27,28,29]. In the present work, we have used this approach to show the most frequent words found in scientific literature on pulmonary rehabilitation before and after COVID-19. 

The lexicon analysis of pulmonary rehabilitation in COVID-19 is discordant with respect to the same analysis of scientific literature in ILD. The term “rehabilitation” has a central role in papers on COVID-19 and is more connected with terms such as “exercise”, “physical”, “treatment”, and “training”. The word “function” appears associated with recovery of motor and daily activities in people with COVID-19, while it is most closely related to the pulmonary physiological response in patients with ILD. The absolute value of the term “rehabilitation” or of other terms with a similar meaning (e.g., exercise) is very low in the scientific literature concerning ILD. Other terms usually applied in the rehabilitation field (e.g., exercise, training, program) are more connected to words that refer to organ function, such as “pulmonary” and “respiratory” [20]. Finally, in both graphs, words indicating research activity (e.g., evidence, research) are very uncommon.

With regard to the clinical practice, the key element for pulmonary rehabilitation is exercise [30]. It is interesting to note that the absolute value of the term “rehabilitation” or words with a similar meaning (i.e., exercise) is very low in the literature on ILD. We hypothesize that this may be related to the lack of structured and tailored training in the management of these patients, which, in turn, could determine a non-homogeneous use of the term “rehabilitation” or similar terms. In addition, we note a clear dissociation between the main concepts related to disability as a consequence of the lung disease and the words used to define it. In particular, there is a paucity of terms designating a classification of functioning, disability, and health core sets for pulmonary diseases [31,32]. 

These differences in patients with COVID-19, as shown in the present study, could be due to the earliest recourse to rehabilitation from intensive care units (ICUs). It should be noted that COVID-19 affects organs and systems much more widely than specific lung diseases such as ILD [22]. This aspect could also influence the clinical care and rehabilitation model, requiring a wider use of rehabilitative treatments than those proposed in ILD. In fact, hospital care settings and timing of hospitalization are very different in the two diseases. The care units involved in the hospitalization of patients with COVID-19 are mostly intensive care units (ICUs) [33,34,35,36]. Data analysis reported that a median length of hospital stay ranged from 4 to 53 days in China and 4 to 21 days outside China [34]. We also believe that these data may be underestimated mainly due to the clinical setting considered. Hospitalized patients with acute exacerbations of ILD are fewer and admitted to less intensive settings (e.g., cardiorespiratory, clinical medicine, rehabilitation units) [37]. A possible reason for this lexical discrepancy could also be related to different geographical areas mainly involved in the rehabilitation research on ILD and COVID-19. With the LENGTH method, the geographic information, based on the first author’s affiliation, can reveal the weight worldwide with respect to the research topic, and the concentration of scientific literature allows for inferring the research interest in a specific field [19]. Most of the scientific literature on rehabilitation in ILD was from researchers from Anglo-Saxon countries (mainly the USA, but also the UK and Australia), with a relevant gap compared to other countries. On the other hand, considering rehabilitation in patients with COVID-19, Chinese researchers were found to be the most active in research activity. Additionally, Italian researchers showed relevant activity in the field of rehabilitation during COVID-19, despite the small number of researchers compared to other countries. These results may be dependent on the fact that these countries were the most and earliest affected countries by the pandemic. 

The study presents some limitations. The first is related to the LENGTH method itself, which only evaluates the frequency of words in the abstracts and in the titles of papers and not in the text. On the other hand, the analysis of titles and abstracts is always possible, even when the full text is not available. In COVID-19, rehabilitation is currently ongoing due to the fact of this disease is too young and variable, with a wide variability of symptoms and very different implications. Hence, rehabilitation could be substantially different in the hospitalized and non-hospitalized patients, depending on the phase (acute/sub-acute/post-acute), and in patients with the long-COVID. Another limitation is that the number of search terms found in the analysis based on the graph theory could be too narrow to scope the whole literature of interest. Although this method only allows for a general lexical evaluation of the considered studies, abstracts reflect the core contents of the paper. Word choice is related to researchers’ experience and should be properly adapted (in number and type) for each lexical analysis. In this study, the results probably reflect the increased interest of researchers about patients with COVID-19. Other possible findings could be determined using other strings of the search terms. Additionally, the type of selected disease (ILD) may have influenced the results. ILD is characterized by a slow functional pulmonary degenerative disease, with huge differences between types for inside interstitium pathologies. This could also affect the rehabilitation. At the same time, our results could be interesting in comparison with patients with long-COVID, who were not taken into account in our study. In further studies, this method could help in the analysis of the different rehabilitation between more acute diseases such as ARDS by different pathologies (COVID-19 vs. no COVID-19 patients).

Then, we did not perform any qualitative/quantitative analysis of the included studies, deviating from the standard literature review method. On the other hand, with this method, we have included a large body of literature. To overcome the possible limitations of the method used in this study, future studies should encompass a systematic review and meta-analysis of selected items to assess the efficacy of the different treatments. An interesting analysis could also concern the components of the exercise prescription (frequency, intensity, time, and type) in the rehabilitative programs. 

Our results need to be corroborated over time and we recommend extreme caution in promoting specific recommendations due to a lack of evidence, especially in frail patients, who are now facing late side effects of acute COVID-19 infection [8,10,38,39]. The specific treatments should also be evaluated in the light of the guidelines, which are continuously updated. Due to the fragile nature of the patients, many other aspects should be considered in the evaluation of future studies, such as hospitalization, polypharmacy, co-morbidity, and coexisting chronic conditions, that should be assessed together with specific symptoms of COVID-19 [40,41,42,43].

## 4. Conclusions

Graph theory offers a new methodological approach to perform a literature analysis, analyzing the connection between terms and diseases to define specific topics. Additionally, it displays, through visual and numerical information, the features of a large amount of data. This represents a remarkable advantage due to high and continuous data evolution. With this study, we aimed to represent the publication trends in respiratory rehabilitation literature on the basis of the lexicon analysis. The information obtained sheds new light on the analysis of scientific literature, revealing new perspectives useful for clinical and research speculation. Our results show a clear discrepancy between rehabilitation proposed for COVID-19 and that for interstitial lung diseases. Although there are only few clinical trials, our results show that rehabilitation in patients with COVID-19 is more connected to exercises and physical training for the recovery of global disability and motor impairments. Conversely, the pulmonary rehabilitation in patients with ILD is more oriented to clinical efficacy due to its connections with words such as “lung”, “disease”, “interstitial”, and “quality”. 

The assessment of the terms used for the description of the rehabilitation approaches may allow a better understanding of the status of the topic. The use of clear and shared words may help the development of common knowledge and avoid literature misunderstandings. Finally, this type of large analysis of literature may lead towards a further person-centered rehabilitation, also facilitating the construction of transdisciplinary models into routine practice worldwide [44].
